# COVID-19 and unfavorable changes in mental health unrelated to changes in physical activity, sedentary time, and health behaviors among Swedish adolescents: A longitudinal study

**DOI:** 10.3389/fpubh.2023.1115789

**Published:** 2023-03-10

**Authors:** Gisela Nyberg, Björg Helgadóttir, Karin Kjellenberg, Örjan Ekblom

**Affiliations:** ^1^Department of Physical Activity and Health, The Swedish School of Sport and Health Sciences (GIH), Stockholm, Sweden; ^2^Department of Global Public Health, Karolinska Institutet, Stockholm, Sweden; ^3^Department of Clinical Neuroscience, Division of Insurance Medicine, Karolinska Institutet, Stockholm, Sweden

**Keywords:** accelerometer, screen time, organized sports, anxiety, health-related quality of life, stress, sleep

## Abstract

**Background:**

The COVID-19 pandemic has had major impact on the daily lives of adolescents. This study examined whether mental health outcomes had changed over the pandemic, and if such changes were related to changes in physical activity (PA), sedentary time, sleep, screen time, and participation in organized sports.

**Materials and methods:**

In this longitudinal study, data were collected in autumn 2019 with follow-up measurements in spring 2021. In total, 558 schools were invited and 34 schools around Stockholm with a variation in socioeconomic background were included. Physical activity and sedentary time were measured for seven consecutive days by accelerometry (Actigraph). Anxiety, health-related quality of life (HRQoL), psychosomatic health, stress, sleep duration, screen time, and organized sports participation were self-reported in questionnaires. Linear models were applied to estimate associations between changes in mental health outcomes and exposures.

**Results:**

From the baseline sample of 1,139 participants, 585 (55% girls), mean (SD) age 14.9 (0.3) years, participated in the follow-up. Between 2019 and 2021, there was a decrease in HRQoL [mean difference −1.7 (−2.3, −1.2), *p* < 0.001], increase in psychosomatic health problems [mean difference 1.8 (1.3, 2.3), *p* < 0.001], and an increase in the number of participants with high stress [from 94 (28%) to 139 (42%), *p* < 0.001]. Weekly light PA and sleep duration decreased and weekly sedentary time and screen time increased unrelated to changes in mental health outcomes. An increase in sleep duration during weekdays was significantly related to both a decrease in anxiety (*B* = −0.71, CI: −1.36, −0.06) and an increase in HRQoL (*B* = 1.00, CI: 0.51, 1.49).

**Conclusion:**

During the COVID-19 pandemic, mental health appears to have been impaired in Swedish adolescents, but unrelated to changes in PA, sedentary time, screen time, or participation in organized sports. However, increased sleep duration on weekdays was related to less anxiety and better HRQoL. The results may help policy makers and other stakeholders comprehend the differential effects of the COVID-19 pandemic on mental health outcomes and help guiding the planning of policy actions.

**Trial registration:**

ISRCTN15689873.

## Introduction

There has been an increase in self-reported mental health and psychosomatic health problems in recent years among Swedish adolescents (1, 2). Globally, psychiatric conditions were the single largest contributor to the overall burden of disease in ages under 20 years in high income countries in 2019 (3). In addition, mental health problems at young age have been associated with an increased risk for prolonged and more severe forms of mental illnesses later in life (4). Importantly, physical activity (PA) has been found to be associated with better mental health outcomes (5). However, the majority of adolescents do not meet the PA recommendation of 60 min of daily PA at moderate to vigorous intensity (MVPA) (6, 7). In addition, sedentary behavior, in particular screen time, has been associated with poor mental health (5).

The COVID-19 pandemic restrictions may have had an influence on current and future mental health among adolescents. It is therefore important to assess the impact of the pandemic on mental health so relevant interventions can be developed to mitigate its effects. In Sweden, restrictions related to the pandemic were minimal compared to those in many other countries. There was no lockdown but there were recommendations to keep physical distance, avoid crowding and large gatherings and non-essential traveling, and to stay home and avoid close contact with others when ill. Organized physical activities continued, albeit with adapted forms of participation, but matches and competitions were not allowed for adolescents. A large proportion of the physical education classes were performed outdoors. There were no school closures for adolescents but most schools had distance learning to some degree.

Global findings on changes in mental health during the pandemic are a cause of concern, suggesting an impact on mental health among adolescents (8, 9). In addition, studies indicate that PA has decreased and sedentary behavior has increased during the pandemic (10, 11). Currently, few longitudinal studies investigating the effects of COVID-19 on adolescents' mental health include baseline data from just before the pandemic. In addition, most previous studies measuring PA have used self-reported measurements (11). To our knowledge, no longitudinal studies have yet been published that have data on mental health outcomes in relation to detailed device-measured PA, sedentary time, and health behaviors just before and during the pandemic.

The first phase of this study was performed between September and December 2019, thus providing unique data from just before the pandemic started. The data include multiple measures of mental health outcomes, device-measured PA, sedentary time, and health behaviors with a mean (SD) follow-up 18.4 (1.0) months later during the pandemic. The aim of this study was therefore to examine potential changes in mental health outcomes [anxiety, health-related quality of life (HRQoL), psychosomatic problems, and stress] and if such changes were related to changes in PA, sedentary time, and health behaviors (sleep, screen time, and participation in organized sports) before and during the COVID-19 pandemic among Swedish boys and girls.

## Materials and methods

### Study population

This longitudinal study used data from the study “Physical activity for healthy brain functions in school youth” (12) collected in September–December 2019 (baseline, *n* = 1,139) and in a follow-up in the same sample (*n* = 585) in April–June 2021.

**Figure 1:**
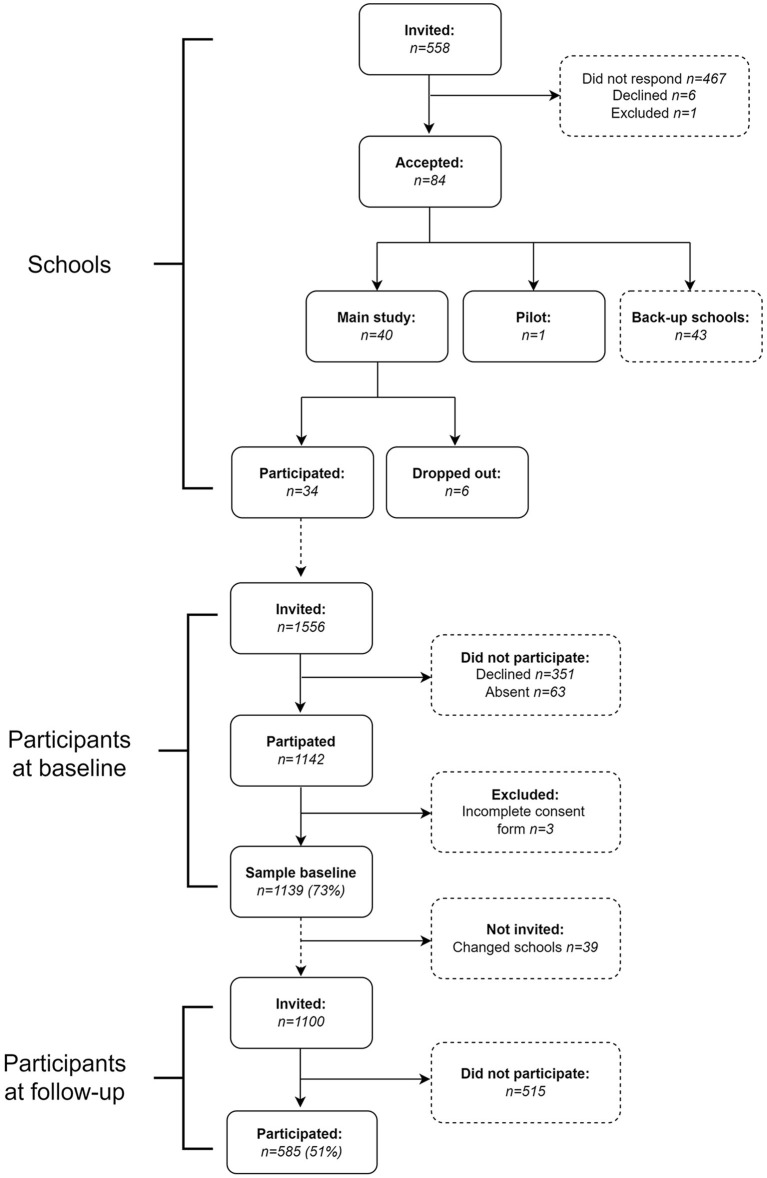
Flow-chart of the schools and participants in the study.

Schools situated within a 2-to-3-h drive from Stockholm, Sweden were invited to participate in the study. Schools with a sports profile (having additional scheduled PA every week), with fewer than 15 students in each class, or with a student population that did not speak Swedish, were excluded. In total, 558 schools with students in grade seven (aged 13–14 years) were invited to participate in the study and 84 schools agreed to participate at baseline (see flowchart, Figure 1). For feasibility reasons, the inclusion stopped after 40 schools had been included. The schools represented a variation in type of municipality (urban and rural), socioeconomic background (parental education), and school organization (independent and public schools). One to four classes participated from each school. All the students in the participating classes were invited to participate in the study. In total, 1,556 students were invited and 1,139 accepted (73% participation rate). In total, 12% of the schools were geographically located in rural areas and 88% in urban areas. The proportion of parents with high education, on school level, was 60%. Eleven schools (32%) were independents schools and 23 schools (68%) were public schools. In Sweden, parents can choose between independent and public schools without paying any fees. At follow-up, schools were contacted again and asked to assist in the data collection. Many schools then had distance education and a high absence due to sickness. A total of 28 schools agreed to participate in the follow-up measures (82%) and the students in the remaining six schools were invited to participate by post or e-mail. In total, 39 participants had moved; 1,100 students were invited, and 585 students agreed to participate (51% participation rate).

### Data collection

At baseline, the measurements were conducted at GIH by trained researchers. Physical activity and sedentary time were measured by accelerometry. Anxiety, health-related quality of life (HRQoL), psychosomatic health, stress, sleep duration, screen time, and sports participation were self-reported in questionnaires. Further methodological details can be found elsewhere (12). At follow-up, accelerometers were sent to schools (*n* = 982) or by post to individual students (*n* = 157). The teacher or student was asked to report the first accelerometer wear-day and the participant wore the accelerometer for seven consecutive days. The accelerometers were sent back in pre-paid envelopes. Information on how to log in to complete the web-based questionnaire was distributed by the teachers or by post. The link was also sent to the e-mail address that the participant had provided at baseline. The teachers provided information regarding the degree of distance learning during the accelerometer wear period. Information from schools that declined to assist in the data collection was provided by the participating students in the questionnaire or by e-mail. The participants received a gift card as a compensation (in total 15€).

At both measurements, the students gave written consent. The study was conducted in accordance with the Declaration of Helsinki and the protocol was approved by the Ethical Review Agency in Stockholm, Sweden (Dnr: 2019-03579 and Dnr: 2021-01235). Trial registration: ISRCTN15689873.

### Outcomes

#### Mental health

Anxiety was measured using a short version of the Spence Children's Anxiety Scale (SCAS-S). The questionnaire includes 19 items and each item is rated on a four-point scale with the options of “never,” “sometimes,” “often,” and “always.” The scores are summed with higher values reflecting more symptoms of anxiety (13).

Health-related quality of life was measured using the questionnaire Kidscreen-10 (14). The scale includes 10 items about how the students have felt during the last week (for example, “fit and well”), and how often. Answers range from “not at all” to “extremely” or from “never” to “always” on a five-point scale. The scores are summed with higher values indicating better well-being.

Psychosomatic health was assessed using the questionnaire Psychosomatic Problems Scale (PSP) (15). The scale includes eight items with questions to the students about how they have felt during the last week, for example if they have had a “headache” or “stomach ache.” Each item is rated on a 5-point Likert-scale, with the options “never,” “seldom,” “sometimes,” “often,” and “always.” The scores are summed with higher values indicating more psychosomatic problems.

Stress was measured with the Single Item Stress Question (SISQ) (16). Participants responded using a 5-point Likert scale with the options “not at all,” “only a little,” “to some extent,” “rather much,” and “very much.”

Changes in the mental health variables were calculated by subtracting the baseline values from the follow-up values. Change in stress was derived by including only those who had low stress at baseline (not at all, only a little, to some extent) and categorizing them as continuing to have low stress or changing to having high stress (rather much, very much).

### Exposures

#### Physical activity and time spent sedentary

Time spent in PA and sedentary was collected during 7 days using hip-worn accelerometers (model GT3X+, Actigraph, LCC, Pensacola, FL, USA). The same method for data collection and data processing was used at baseline and at follow-up described in more detail elsewhere (12). The program ActiLife, version 6.13.3 was used to process data from the accelerometers. Individual wake and sleep times from the questionnaire were used to define time awake. Days with wear-time of at least 500 min were defined as valid days and at least three valid days, where at least one of those days was a weekend day, were included in the weekly analyses. The total wear-time from the monitors was categorized into sedentary intensity (0–100 counts per min), light PA intensity (101–2,295 counts per min), and MVPA (>2,295 counts per min) (17). The results are presented as minutes within the respective intensity, as averages per day over the whole week. Change in PA and sedentary time was calculated by subtracting the baseline values from the follow-up values.

#### Sleep

Sleep duration was calculated using bed-time and time for getting up reported by the participants in the questionnaire for weekdays and weekends. The response alternatives were in 30-min intervals. Change in sleep duration was calculated in hours.

#### Screen time

The amount of screen time outside school hours (not including schoolwork) on weekdays and weekends was self-reported by the participants. The response alternatives were: no time at all, < 1 h, 1–2 h, 3–4 h, 5–6 h, and 7 h or more, and were categorized into ≤ 2 h, 3–4 h, and ≥5 h. The responses from baseline and follow-up were coded to reflect either an increase or decrease in screen time or no change in screen time.

#### Organized sports participation

The participants reported in the questionnaire if they were active in any sports organization (yes/no). The answers from baseline and follow-up were combined to reflect change in organized sports: (1) no change, i.e., either participated at both baseline and follow-up or did not participate at both baseline and follow-up, (2) dropped out, i.e., participated in organized sports at baseline but not at follow-up, and (3) started, i.e., did not participate in organized sports at baseline but participated at follow-up.

#### Parental education and country of birth

Register data on parental education was collected from the Statistics of Sweden and dichotomized into ≤ 12 years (low) and >12 years (high) of education. The highest level of education attained by either of the parents was used. The participant's and their parents' country of birth were reported by the participants in the questionnaire. Parents' country of birth was classified as “both born in Sweden,” “one born outside Sweden and one born in Sweden,” and “both parents born outside Sweden.”

#### Anthropometry

Baseline data was used on body mass index (BMI). Underweight, normal weight, overweight and obesity were defined according to the International Obesity Task Force recommendations, with different cut-off values depending on age and sex (18).

#### Distance learning

The amount of distance learning was self-reported at follow-up by the participants by asking them “Did you have distance learning during January to April 2021?” The response alternatives were “no, not at all,” “yes partially distance,” and “yes fully distance.” The two last alternatives were combined as very few (*n* = 27) reported full distance learning.

### Statistical analysis

Descriptive statistics are presented as percentages or means with standard deviations. Differences between girls and boys for the background variables were tested with chi-square tests and independent *t*-tests for categorical and continuous variables, respectively. The same analyses were used for comparing those who participated both at baseline and follow-up and those who did not participate at follow-up. Changes in mental health outcomes, as well as PA, sedentary time, sleep, screen time, and organized sports were analyzed with McNemars change test for categorical variables and paired *t*-tests for continuous variables. Relationships between the exposure variables and the continuous outcome variables (change in anxiety, HRQoL, and psychosomatic health) were analyzed in separate models with ANCOVAs (analysis of covariance), reported as non-standardized betas with 95% confidence intervals. The binary outcome (change in stress) was modeled using multivariable logistic regression models, reported as odds ratios with 95% confidence intervals. As the outcome variables showed minimal influence of clusters (0–1%), all the models were on one level. The independent variables included in the models were checked for multi-collinearity, but none was detected. Homoscedasticity assumptions were checked through plotting of residuals against predicted values and all residuals were visually checked for normality of distribution. All models were adjusted for gender, parental education, parental country of birth as well as the baseline value of the outcome variable. Wear time was added in the initial analyses and did not alter the results and was therefore not included in the final analyses. No imputations for missing data or loss to follow-up were performed. All statistical tests were two-sided with a significance threshold of *P* < 0.05. The data analyses were performed in IBM SPSS Statistics, version 27 (IBM Corp., Armonk, N.Y., USA).

## Results

### Sample characteristics

From the baseline sample of 1,139 participants, 585 (51%) participated in the follow-up measurements. The mean (SD) age was 14.9 (0.3) years and 263 were boys (45%) and 321 girls (55%), one participant did not reveal gender. There were significant differences between the baseline and follow-up in gender composition, the proportions of parental country of birth, and the proportions of stress, as shown in Table 1. There were no significant differences between the baseline and follow-up samples in the other variables.

**Table 1:** Baseline characteristics of participants and drop-outs.

**Variables measured at baseline**	**Participants**	**Drop-outs**	***p*-Value^a^**
No. (%) with data^b^	585 (51.4)	554 (48.6)	
**Gender [No. (%)]**			
Boys	263 (45.0)	295 (53.2)	**0.006**
Girls	321 (55.0)	259 (46.8)	
**Parental education [No. (%)]**			
Low ( ≤ 12 years)	389 (68.1)	341 (64.2)	0.17
High (>12 years)	182 (31.9)	190 (35.8)	
**Participant country of birth [No. (%)]**			
Sweden	501 (86.1)	466 (85.2)	0.86
Europe	24 (4.1)	22 (4.0)	
Outside Europe	57 (9.8)	59 (10.8)	
**Parental country of birth [No. (%)]**			
Both parents born in Sweden	357 (62.5)	299 (55.8)	**0.008**
One born outside Sweden	69 (12.1)	99 (18.5)	
Both born outside Sweden	145 (25.4)	138 (25.7)	
**BMI status [No. (%)]**			
Underweight and normal weight	467 (80.2)	437 (79.0)	0.61
Overweight and obesity	115 (19.8)	116 (21.0)	
**Mental health [mean (SD)]**			
Anxiety (SCAS-S) (Missing *n* = 66)	13.9 (7.7)	13.4 (8.1)	0.23
HRQoL (Kidscreen) (Missing *n* = 42)	39.6 (5.2)	39.6 (5.6)	0.96
Psychosomatic problems (PSP) (Missing *n* = 43)	17.3 (5.2)	17.3 (5.7)	0.88
**Stress [No. (%)]**			
Not at all	83 (14.4)	123 (23.1)	**0.002**
Slightly	247 (42.7)	201 (37.7)	
Somewhat	131 (22.7)	101 (18.9)	
Quite much	88 (15.2)	72 (13.5)	
Very much	29 (5.0)	36 (6.8)	
**Moderate to vigorous physical activity [mean (SD)]**			
Week (min/day) (Missing *n* = 236)	52.3 (19.1)	51.6 (18.9)	0.63
**Light physical activity [mean (SD)]**			
Week (min/day) (Missing *n* = 236)	140.3 (29.1)	136.7 (31.4)	0.07
**Sedentary [mean (SD)]**			
Week (min/day) (Missing *n* = 236)	603.7 (62.5)	600.1 (70.9)	0.43
**Sleep [mean (SD)]**			
Weekdays (h) (Missing *n* = 17)	8.6 (0.9)	8.5 (1.0)	0.47
Weekends (h) (Missing *n* = 17)	9.8 (1.4)	10.0 (1.4)	0.19
**Screen time (weekdays) [No. (%)]**			0.78
≤ 2 h	190 (32.7)	170 (31.2)	
3–4 h	260 (44.8)	255 (46.8)	
≥5 h	131 (22.5)	120 (22.0)	
**Screen time (weekends) [No. (%)]**			0.44
≤ 2 h	84 (14.5)	94 (17.2)	
3–4 h	213 (36.9)	199 (36.5)	
≥5 h	281 (48.6)	252 (46.2)	

HRQoL, health-related quality of life. Results in bold are significant at α < 0.05.

^a^Chi square test for categorical variables and independent sample *t*-test for continuous variables.

^b^One participant was non-binary and was therefore not included in the analyses stratified by gender.

### Change in mental health outcomes

Between 2019 and 2021, there was a decrease in HRQoL [mean difference −1.7 (−2.3, −1.2), *p* < 0.001], increase in psychosomatic health problems [mean difference 1.8 (1.3, 2.3), *p* < 0.001], and an increase in the number of participants with high stress [from 94 (28%) to 139 (42%), *p* < 0.001], in the total sample and also among both boys and girls when stratified (see Table 2). There was no significant change in anxiety in the total sample but among girls there was a significant increase [mean difference 1.7 (0.6, 2.7), *p* = 0.001], and among boys a significant decrease [mean difference −1.2 (−2.1, −0.1), *p* = 0.005], between baseline and follow-up measurements.

**Table 2:** Baseline and follow-up data for mental health outcomes, stratified by gender.

		**All participants**					**Girls**					**Boys**			
		**Baseline**	**Follow-up**				**Baseline**	**Follow-up**				**Baseline**	**Follow-up**		
		**(2019)**	**(2021)**				**(2019)**	**(2021)**				**(2019)**	**(2021)**		
	***n***			**Mean difference (95% CI)**	***p-*** **value**	***n***			**Mean difference (95% CI)**	***p*** **-value**	***n***			**Mean difference (95% CI)**	***p*** **-value**
Anxiety^a^, Mean (SD)	457	13.9 (7.9)	14.2 (9.6)	0.3 (−0.4, 1.0)	0.35	248	16.1 (8.0)	17.8 (9.3)	**1.7 (0.6, 2.7)**	**0.001**	208	11.2 (6.9)	9.9 (8.1)	**−1.2 (−2.1**, **−0.1)**	**0.005**
HRQoL^a^ Mean (SD)	438	39.8 (5.2)	38.1 (5.2)	**−1.7 (−2.3**, **−1.2)**	**< 0.001**	233	38.5 (5.1)	36.6 (5.9)	**−2.0 (−2.7**, **−1.2)**	**< 0.001**	204	41.3 (4.9)	39.8 (5.7)	**−1.5 (−2.3**, **−0.6)**	**< 0.001**
Psychosomatic problems^a^ Mean (SD)	501	9.3 (5.3)	11.1 (6.7)	**1.8 (1.3, 2.3)**	**< 0.001**	277	10.7 (5.3)	13.4 (6.6)	**2.7 (2.0, 3.4)**	**< 0.001**	223	7.5 (4.6)	8.2 (5.8)	**0.7 (0.0, 1.4)**	**0.049**
Stress^b^															
Low stress [No. (%)]	333	239 (71.8)	194 (58.3)	N/A	**< 0.001**	178	101 (56.7)	69 (38.8)	N/A	**< 0.001**	154	138 (89.6)	125 (81.2)	N/A	**< 0.001**
High stress [No. (%)]		94 (28.2)	139 (41.7)	N/A			77 (43.3)	109 (61.2)	N/A			16 (10.4)	29 (18.8)	N/A	

HRQoL, health-related quality of life. One participant was non-binary and was therefore not included in the analyses stratified by gender. Results in bold are significant at α < 0.05.

^a^Changes from baseline to follow-up tested with paired *t*-tests.

^b^Changes from baseline to follow-up tested with McNemar change test.

### Change in physical activity, sedentary time, and health behaviors

In total, 264 participants had valid accelerometer registrations for the whole week, both at baseline and follow-up. There were no significant differences between participants with and without valid accelerometer registrations in anxiety (*p* = 0.31), HRQoL (*p* = 0.11), psychosomatic problems (*p* = 0.11), and stress (*p* = 0.44). Accelerometer wear-time at baseline compared to follow-up was higher for all participants, mean (SD) 803 (56) min/791 (71) min, *p* = 0.007 and girls 806 (55) min/793 (77) min, *p* = 0.02.

Between baseline and follow-up measurements, there was a significant decrease of an average of 20 min/day in light PA for the whole sample (*p* < 0.001), for girls (~18 min, *p* < 0.001), and for boys (~23 min, *p* < 0.001) but there was no significant change in MVPA, as shown in Table 3. There was a significant increase of an average of ~8 min in sedentary time for the whole group (*p* = 0.04) and for boys (~16 min, *p* = 0.03).

**Table 3:** Baseline and follow-up data for physical activity, sedentary time, and health behaviors, stratified by gender.

		**All participants**				**Girls**				**Boys**		
		**Baseline**	**Follow-up**			**Baseline**	**Follow-up**			**Baseline**	**Follow-up**	
		**(2019)**	**(2021)**			**(2019)**	**(2021)**			**(2019)**	**(2021)**	
	***n***			***p*** **-value**	***n***			***p*** **-value**	***n***			***p-*** **value**
**MVPA in min** ^a^												
Week, mean (SD)	264	51.7 (19.0)	51.2 (23.5)	0.75	166	50.0 (18.1)	49.9 (22.6)	0.97	97	54.8 (20.1)	53.8 (24.7)	0.69
**Light PA in min** ^a^												
Week, mean (SD)	264	139.9 (28.4)	119.9 (31.5)	**< 0.001**	166	138.5 (27.4)	120.2 (29.7)	**< 0.001**	97	142.5 (30.1)	119.9 (34.1)	**< 0.001**
**Sedentary in min** ^a^												
Week, mean (SD)	264	611.3 (60.8)	619.6 (70.7)	**0.04**	166	617.8 (59.3)	622.4 (69.3)	0.37	97	599.7 (62.0)	615.6 (73.4)	**0.03**
**Sleep in h** ^a^												
Weekdays, mean (SD)	557	8.6 (0.9)	8.1 (1.0)	**< 0.001**	310	8.5 (0.9)	8.0 (1.0)	**< 0.001**	246	8.6 (0.9)	8.3 (0.9)	**< 0.001**
Weekends, mean (SD)	558	9.8 (1.4)	9.5 (1.3)	**< 0.001**	311	9.9 (1.3)	9.5 (1.3)	**< 0.001**	246	9.8 (1.5)	9.5 (1.2)	**0.02**
**Screen time during weekdays**^b^ **[No. (%)]**												
< 1 h per day	556	39 (7.0)	12 (2.2)	**< 0.001**	310	19 (6.1)	5 (1.6)	**< 0.001**	245	20 (8.2)	7 (2.9)	**< 0.001**
1–2 h per day		144 (25.9)	88 (15.8)			77 (24.8)	41 (13.2)			66 (26.9)	47 (19.2)	
3–4 h per day		245 (44.1)	231 (41.5)			144 (46.5)	132 (42.6)			101 (41.2)	99 (40.4)	
5–6 h per day		101 (18.2)	177 (31.8)			55 (17.7)	110 (35.5)			46 (18.8)	66 (26.9)	
7 h or more		27 (4.9)	48 (8.6)			15 (4.8)	22 (7.1)			12 (4.9)	26 (10.6)	
**Screen time during weekends**^b^ **[No. (%)]**												
< 1 h per day	553	18 (3.3)	10 (1.8)	**< 0.001**	310	8 (2.6)	6 (1.9)	**< 0.001**	242	10 (4.1)	4 (1.7)	**< 0.001**
1–2 h per day		63 (11.4)	29 (5.2)			32 (10.3)	14 (4.5)			31 (12.8)	15 (6.2)	
3–4 h per day		201 (36.3)	140 (25.3)			123 (39.7)	73 (23.5)			77 (31.8)	66 (27.3)	
5–6 h per day		174 (31.5)	214 (38.7)			100 (32.3)	127 (41.0)			74 (30.6)	87 (36.0)	
7 h or more		97 (17.5)	160 (28.9)			47 (15.2)	90 (29.0)			50 (20.7)	70 (28.9)	
**Organized sport participation**^b^ **[No. (%)]**												
No	515	134 (26.0)	128 (24.9)	0.54	288	73 (25.3)	65 (22.6)	0.26	227	61 (26.9)	63 (27.8)	0.77
Yes		381 (74.0)	387 (75.1)			215 (74.7)	223 (77.4)			166 (73.1)	164 (72.2)	
**Distance learning spring 2021**^c^ **[No. (%)]**												
No	551	N/A	118 (21.4)	N/A	306	N/A	66 (21.6)	N/A	244	N/A	52 (21.3)	N/A
Yes		N/A	433 (78.6)			N/A	240 (78.4)			N/A	192 (78.7)	

MVPA, moderate to vigorous physical activity; PA, physical activity. One participant was non-binary and was therefore not included in the analyses stratified by gender. Results in bold are significant at α < 0.05.

^a^Changes from baseline to follow-up tested with paired *t*-tests.

^b^Changes from baseline to follow-up tested with Wilcoxon tests for paired samples.

^c^Only measured at follow-up.

Sleep duration decreased significantly during weekdays and weekends for the whole group (~0.5 h/night, *p* < 0.001)/(~0.3 h/night, *p* < 0.001), and also significantly among girls and boys when stratified (see Table 3).

Screen time increased significantly between baseline and follow-up: for example, the proportion of participants reporting ≥5 h of screen time during weekdays increased from 23.1% to 40.4% (girls 22.5% to 42.6% and boys 23.7% to 37.5%) and during weekends from 49% to 67.6% (girls 47.5% to 70% and boys 51.3% to 64.9%).

There was no significant difference between baseline and follow-up in organized sports participation.

At follow-up measurements, 433 participants (78.6%) reported that they had some amount of distance learning during the spring period.

### Relationships between changes in mental health outcomes and changes in physical activity, sedentary time, and health behaviors

Results from the multivariable logistic regression are presented in Table 4 and showed that an increase in sleep during weekdays was significantly related to both a decrease in anxiety (*B* = −0.71, CI: −1.36, −0.06) and an increase in HRQoL (*B* = 1.00, CI: 0.51, 1.49). No significant relationships were found between changes in mental health outcomes and changes in MVPA, light PA, sedentary time, organized sports participation, and screen time.

**Table 4:** Relationships between changes in physical activity, sedentary time, and health behaviors, from baseline to follow-up.

	**Change in anxiety (SCAS-S)**		**Change in HRQoL (Kidscreen)**		**Change in psychosomatic problems (PSP)**		**Change in stress (SISQ)**	
	β **(95% CI)**	***p***	β **(95% CI)**	***p***	β **(95% CI)**	***p***	**OR (95% CI)**	***p***
**Change in MVPA**								
Week (min/day)	−0.02 (−0.05, 0.02)	0.41	0.01 (−0.02, 0.04)	0.53	−0.00 (−0.03, 0.02)	0.84	0.99 (0.98, 1.01)	0.46
**Change in light PA**								
Week (min/day)	−0.01 (−0.04, 0.02)	0.74	0.02 (−0.00, 0.04)	0.09	0.00 (−0.02, 0.02)	0.98	1.00 (0.99, 1.01)	0.99
**Change in sedentary**								
Week (min/day)	−0.00 (−0.02, 0.01)	0.58	−0.00 (−0.01, 0.01)	0.53	−0.00 (−0.01, 0.01)	0.68	1.00 (0.99, 1.00)	0.69
**Change in sleep**								
Weekdays (h)	–**0.71 (**–**1.36**, –**0.06)**	**0.03**	**1.00 (0.51, 1.49)**	**< 0.001**	−0.40 (−0.89, 0.09)	0.10	0.95 (0.74, 1.22)	0.68
Weekends (h)	−0.23 (−0.66, 0.21)	0.30	0.26 (−0.06, 0.59)	0.11	−0.24 (−0.55, 0.08)	0.14	0.88 (0.75, 1.03)	0.10
**Change in organized sport participation**								
No change	REF		REF		REF		REF	
Dropped out	1.94 (−0.42, 4.30)	0.11	−1.76 (−3.72, 0.19)	0.08	1.56 (−0.20, 3.31)	0.08	0.88 (0.37, 2.10)	0.77
Started	−0.01 (−2.30, 2.27)	0.99	−0.67 (−2.57, 1.23)	0.49	−0.80 (−2.49, 0.89)	0.35	0.96 (0.41, 2.34)	0.93
**Change in screen time during weekdays**								
No change	REF		REF		REF		REF	
Increase	−0.17 (−1.60, 1.27)	0.82	0.63 (−0.44, 1.70)	0.25	0.03 (−1.03, 1.09)	0.96	1.00 (0.59, 1.68)	0.99
Decrease	0.12 (−1.84, 2.09)	0.90	0.39 (−1.15, 1.93)	0.62	0.31 (−1.20, 1.82)	0.68	1.82 (0.90, 3.70)	0.10
**Change in screen time during weekends**								
No change	REF		REF		REF		REF	
Increase	−0.71 (−2.15, 0.73)	0.33	0.67 (−0.44, 1.78)	0.23	−0.72 (−1.80, 0.35)	0.19	0.81 (0.49, 1.36)	0.43
Decrease	−0.30 (−2.30, 1.71)	0.77	0.02 (−1.52, 1.57)	0.98	−1.32 (−2.80, 0.17)	0.08	1.19 (0.59, 2.43)	0.63
**Distance learning spring 2021** ^a^								
No	REF		REF		REF		REF	
Yes	−0.71 (−2.31, 0.89)	0.39	0.61 (−0.67, 1.88)	0.35	−0.06 (−1.26, 1.15)	0.93	0.79 (0.45, 1.41)	0.43

MVPA, moderate to vigorous physical activity; PA, physical activity. Results in **bold** are significant at α < 0.05. All models are adjusted for gender, parental education, parental country of birth, and baseline values of the outcome.

^a^Measured at follow-up.

## Discussion

To the best of our knowledge, this is the first longitudinal study with data on mental health outcomes in relation to detailed device-measured PA, sedentary time, and health behaviors, just before and during the pandemic. Our findings show that self-reported HRQoL, psychosomatic health, and stress have worsened in adolescents in Sweden during the COVID-19 pandemic but unrelated to changes in PA, sedentary time, screen time, or participation in organized sports. Nonetheless, an increase in sleep duration during weekdays was related to better mental health.

The change in mental health in this study is in line with studies in two systematic reviews showing that the COVID-19 pandemic had impacted the mental health of adolescents (8, 9). Studies from Norway (*n* = 3,752) (19) and Australia (*n* = 248) (20) also found that anxiety and depressive symptoms increased between baseline and follow-up in adolescents. In contrast, one Swedish study found no longitudinal changes in mental health, PA or health behaviors among adolescents (*n* = 584) (21). Similar results have also been reported in two studies of adolescents in China where no deterioration in adolescents' mental health was found (22, 23). Studies in England have shown conflicting findings: some data indicated increased depressive symptoms and other data indicated no changes in anxiety or wellbeing (24). The overall variability of the results between studies might relate to different ages and cultural contexts of participants. In addition, differences in the scope of pandemic restrictions, different study designs, outcome measures, heterogeneity in methods, and length of follow-up periods may have played a role.

The observational design prevented any control group. Therefore, the mean change in the prevalence of mental health in this study cannot be fully attributed to the COVID-19 pandemic, as no comparable data are available. However, the main question was to investigate if any such changes were related to changes in PA variables. The main result from these analyses were that only few such relations were found. To what extent these findings can be relevant for other situations, i.e., without a pandemic, is difficult to assess. However, as we identify temporal changes between baseline and follow-up but only few relations to changes in PA, it can be hypothesized that smaller changes in mental health (possibly found in non-pandemic situations) would also be unrelated to changes in PA.

Several studies have shown that the impact on mental health outcomes has been worse for females compared to males (8, 20, 23). In our study sample, the prevalence of mental health problems was higher in girls than in boys but the decrease in well-being as well as impaired psychosomatic health and stress was evident in both boys and girls. However, there was a difference in anxiety where anxiety levels decreased in boys and increased in girls during the pandemic.

The findings in this study showed no decline in MVPA. However, there was a decline in light PA and an increase in sedentary time. In addition, there was an increase in screen time and no change in participation in organized sports. This might imply that Swedish adolescents have not stopped participating in organized activities outside school hours but may be moving less in their everyday lives since they spent more time at home due to distance education. Studies from other countries showed declines in PA levels and increases in sedentary behavior during the pandemic (10, 11).

This study also showed a decrease in sleep duration during the pandemic. Furthermore, sleep duration during weekdays was related to mental health outcomes, suggesting that an increase in sleep duration during weekdays was related to better HRQoL and less anxiety. Other studies that have measured sleep before and during the pandemic showed mixed results, including increased sleep duration (11), fewer sleep problems (23), and no changes in sleep (21) among adolescents.

In a study with Chinese children and adolescents, it was reported that longer screen time before and during the pandemic was associated with a higher risk of psychological symptoms (25). In the present study the results showed no relationships between changes in mental health and changes in PA, sedentary time, screen time, or participation in organized sports, which suggest that there were other factors underlying the negative changes in mental health outcomes. Physical activity did not decline to the same extent in Sweden compared to other countries, possibly explained by Sweden's milder pandemic restrictions. One Swedish study showed that during the pandemic both children and adolescents expressed worries, for example about the disease and death among their relatives, about the future, or missing out on their youth and employment (26). Swedish adolescents further reported increased conflicts with parents, less time spent with peers, and poorer control over their everyday life (27). Similar data are available from Australia (20).

The major strength of this study is the longitudinal design with baseline data collected just before the pandemic in late 2019 and follow-up in the spring of 2021. Another strength is the detailed device-measured PA in relation to several mental health outcomes. One limitation was the loss of valid accelerometer measurements due to insufficient wear time. However, there were no significant differences between participants with and without valid accelerometer measurements in mental health outcomes. Another limitation of the study is that the sample was non-representative and therefore caution with generalizability should be taken. In addition, the drop-out rate between baseline and follow-up might also have impacted the generalizability of the study. However, the recruitment of schools was based on a variation in type of municipality and socioeconomic background of parents and attrition bias may be limited as the results showed few differences in variables between participants and drop-outs. Another limitation, as mentioned above, is that the effects on mental health outcomes might have been confounded by an expected age-related increase in mental health problems within this group and this could have led to a slight overestimation of the changes in mental health outcomes.

## Conclusion

The results suggest that the COVID-19 pandemic has impaired the mental health of Swedish adolescents and that the decrease in mental health was not related to changes in PA, sedentary time, screen time, or participation in organized sports. However, increased sleep duration during weekdays was related to positive changes in anxiety and HRQoL. The results of this study may help policy makers and other stakeholders comprehend the differential effects of the COVID-19 pandemic on mental health outcomes and help guiding the planning of policy actions.

## Data availability statement

The datasets are not available for download to protect the confidentiality of the participants. The data are held at The Swedish School of Sport and Health Sciences. Anonymous data for meta-analyses can be provided upon request from the corresponding author. Requests to access the datasets should be directed to gisela.nyberg@gih.se.

## Ethics statement

The studies involving human participants were reviewed and approved by the Ethical Review Agency in Stockholm, Sweden (Dnr: 2019-03579 and Dnr: 2021-01235). Written informed consent to participate in this study was provided by the participants' legal guardian/next of kin.

## Author contributions

GN, BH, and KK conducted the data collection. BH and KK cleaned, processed, accessed, and verified the data. GN, BH, and ÖE selected the design of the statistical model. BH performed the analyses. GN drafted the manuscript. All authors contributed to the design of the study and the interpretation of the results. All authors contributed to the article and approved the submitted version.

## References

[1] FORTE. Psykiskt Välbefinnande, Psykiska Besvär och Psykiatriska Tillstånd hos Barn och Unga – Begrepp, Mätmetoder och Förekomst (Mental Well-Being, Mental Distress and Mental Disorders among Children, and Young Adults. Terminology, Measurement Methods and Prevalence – An Overview). Stockholm: FORTE (2021).

[2] Public Health Agency of SwedenSkolbarns Hälsovanor i Sverige 2017/2018 (Health Behaviour in School-Aged Children in Sweden 2017/18). Stockholm: Public Health Agency of Sweden (2018).

[3] Institute for Health Metrics Evaluation. Global Burden of Disease (GBD). (2021). Available online at: http://www.healthdata.org/gbd

[4] The National Board of Health and Welfare. Utvecklingen av Psykisk Ohälsa Bland Barn och Unga Vuxna - Till och Med 2016 (Development of Mental Ill-Health Among Children and Youth Until 2016). Stockholm: National Board of Health and Welfare (2017).

[5] Chaput JP Willumsen J Bull F Chou R Ekelund U Firth J. 2020 WHO guidelines on physical activity and sedentary behaviour for children and adolescents aged 5–17 years: summary of the evidence. Int J Behav Nutr Phys Act. (2020) 17:141. 10.1186/s12966-020-01037-z 33239009 PMC7691077

[6] Guthold R Stevens GA Riley LM Bull FC. Global trends in insufficient physical activity among adolescents: a pooled analysis of 298 population-based surveys with 16 million participants. Lancet Child Adolesc Health. (2020) 4:23–35. 10.1016/S2352-4642(19)30323-2 31761562 PMC6919336

[7] Nyberg G Kjellenberg K Froberg A Lindroos AK. A national survey showed low levels of physical activity in a representative sample of Swedish adolescents. Acta Paediatr. (2020) 109:2342–53. 10.1111/apa.15251 32266736

[8] Samji H Wu J Ladak A Vossen C Stewart E Dove N. Review: Mental health impacts of the COVID-19 pandemic on children and youth - a systematic review. Child Adolesc Ment Health. (2022) 27:173–89. 10.1111/camh.12501 34455683 PMC8653204

[9] Newlove-Delgado T Russell AE Mathews F Cross L Bryant E Gudka R. Annual Research Review: The impact of Covid-19 on psychopathology in children and young people worldwide: systematic review of studies with pre- and within-pandemic data. J Child Psychol Psychiatry. (2022) 2022:1–30. 10.1111/jcpp.13716 36421049 PMC10952503

[10] Stockwell S Trott M Tully M Shin J Barnett Y Butler L. Changes in physical activity and sedentary behaviours from before to during the COVID-19 pandemic lockdown: a systematic review. BMJ Open Sport Exerc Med. (2021) 7:e000960. 10.1136/bmjsem-2020-000960 34192010 PMC7852071

[11] Paterson DC Ramage K Moore SA Riazi N Tremblay MS Faulkner G. Exploring the impact of COVID-19 on the movement behaviors of children and youth: a scoping review of evidence after the first year. J Sport Health Sci. (2021) 10:675–89. 10.1016/j.jshs.2021.07.001 34237456 PMC8687706

[12] Nyberg G Ekblom O Kjellenberg K Wang R Larsson H Thedin Jakobsson B. Associations between the school environment and physical activity pattern during school time in swedish adolescents. Int J Environ Res Public Health. (2021) 18:10239. 10.3390/ijerph181910239 34639539 PMC8507782

[13] Ahlen J Vigerland S Ghaderi A. Development of the Spence Children's Anxiety Scale-Short Version (SCAS-S). J Psychopathol Behav Assess. (2018) 40:288–304. 10.1007/s10862-017-9637-3 29937623 PMC5978831

[14] Ravens-Sieberer U Erhart M Rajmil L Herdman M Auquier P Bruil J. Reliability, construct and criterion validity of the KIDSCREEN-10 score: a short measure for children and adolescents' well-being and health-related quality of life. Qual Life Res. (2010) 19:1487–500. 10.1007/s11136-010-9706-5 20668950 PMC2977059

[15] Hagquist C. Psychometric properties of the psychosomatic problems scale: a rasch analysis on adolescent data. Soc Indic Res. (2008) 86:511–23. 10.1007/s11205-007-9186-3

[16] Elo A-L Leppänen A Jahkola A. Validity of a single-item measure of stress symptoms. Scand J Work Environ Health. (2003) 29:444–51. 10.5271/sjweh.752 14712852

[17] Evenson KR Catellier DJ Gill K Ondrak KS McMurray RG. Calibration of two objective measures of physical activity for children. J Sports Sci. (2008) 26:1557–65. 10.1080/02640410802334196 18949660

[18] Cole TJ Lobstein T. Extended international (IOTF) body mass index cut-offs for thinness, overweight and obesity. Pediatr Obes. (2012) 7:284–94. 10.1111/j.2047-6310.2012.00064.x 22715120

[19] Hafstad GS Saetren SS Wentzel-Larsen T Augusti EM. Adolescents' symptoms of anxiety and depression before and during the Covid-19 outbreak - a prospective population-based study of teenagers in Norway. Lancet Reg Health Eur. (2021) 5:100093. 10.1016/j.lanepe.2021.100093 34557820 PMC8454857

[20] Magson NR Freeman JYA Rapee RM Richardson CE Oar EL Fardouly J. Risk and protective factors for prospective changes in adolescent mental health during the COVID-19 pandemic. J Youth Adolesc. (2021) 50:44–57. 10.1007/s10964-020-01332-9 33108542 PMC7590912

[21] Chen Y Osika W Henriksson G Dahlstrand J Friberg P. Impact of COVID-19 pandemic on mental health and health behaviors in Swedish adolescents. Scand J Public Health. (2022) 50:26–32. 10.1177/14034948211021724 34100665 PMC8808000

[22] Lu P Yang L Wang C Xia G Xiang H Chen G. Mental health of new undergraduate students before and after COVID-19 in China. Sci Rep. (2021) 11:18783. 10.1038/s41598-021-98140-3 34552105 PMC8458482

[23] Li Y Zhou Y Ru T Niu J He M Zhou G. How does the COVID-19 affect mental health and sleep among Chinese adolescents: a longitudinal follow-up study. Sleep Med. (2021) 85:246–58. 10.1016/j.sleep.2021.07.008 34388503 PMC8418314

[24] Newlove-Delgado T McManus S Sadler K Thandi S Vizard T Cartwright C. Child mental health in England before and during the COVID-19 lockdown. Lancet Psychiatry. (2021) 8:353–4. 10.1016/S2215-0366(20)30570-8 33444548 PMC8824303

[25] Xiang M Liu Y Yamamoto S Mizoue T Kuwahara K. Association of changes of lifestyle behaviors before and during the COVID-19 pandemic with mental health: a longitudinal study in children and adolescents. Int J Behav Nutr Phys Act. (2022) 19:92. 10.1186/s12966-022-01327-8 35883177 PMC9321278

[26] Sarkadi A Sahlin Torp L Perez-Aronsson A Warner G. Children's expressions of worry during the COVID-19 pandemic in Sweden. J Pediatr Psychol. (2021) 46:939–49. 10.1093/jpepsy/jsab060 34383921 PMC8376257

[27] Kapetanovic S Gurdal S Ander B Sorbring E. Reported changes in adolescent psychosocial functioning during the COVID-19 outbreak. Adolescents. (2021) 1:10–20. 10.3390/adolescents1010002

